# Two new species and new records of the genus *Pinopellis* Assing, 2022 (Coleoptera, Staphylinidae, Paederinae) from China

**DOI:** 10.3897/zookeys.1280.192655

**Published:** 2026-05-26

**Authors:** Lei Huang, Nima Danzeng, Zhong Peng

**Affiliations:** 1 Department of Biology, Shanghai Normal University, Shanghai, 200234, China Department of Biology, Shanghai Normal University Shanghai China; 2 Institute of Plateau Biology of Xizang Autonomous Region, Lhasa, Xizang Autonomous Region, 850001, China Dawa Innovation Studio, Institute of Plateau Biology of Xizang Autonomous Region Lhasa China; 3 Dawa Innovation Studio, Institute of Plateau Biology of Xizang Autonomous Region, Lhasa, Xizang Autonomous Region, 850000, China Institute of Plateau Biology of Xizang Autonomous Region Lhasa China

**Keywords:** Distribution, East Asia, morphology, Pinophilina, rove beetles, taxonomic key

## Abstract

New morphological, taxonomic, and faunistic data on the genus *Pinopellis* Assing, 2022 from China are provided. Two new species of *Pinopellis* are described: *P. liaoi***sp. nov**. (Guangdong: Shaoguan) and *P. yini***sp. nov**. (Xizang: Medog). *Pinopellis biexcisa* Assing, 2022 is recorded from China for the first time. New provincial records are provided for *P. nigripes* (Cameron, 1914) from Fujian, Guangdong, Guangxi, and Xizang, and for *P. yunnanica* Assing, 2022 from Guangdong. Two unidentified species were recorded, and the previously unknown female of *P. yunnanica* is described for the first time. A key to the *Pinopellis* species from China is given.

## Introduction

The genus *Pinopellis* Assing, 2022 is a small genus endemic to East Asia. Currently, the genus includes 23 species in the east Palaearctic and Oriental regions with four species from China (Assing 2022; Li et al. 2026). *Pinopellis* can be readily distinguished from related genera primarily by the complete reduction of the parameres of the aedeagus, a condition otherwise known only in *Phinopilus* Backwelder, 1952, *Pinonepalus* Coiffait, 1982, and the markedly different *Pinocharis* Fagel, 1963. It differs from *Phinopilus* by the slenderer antennae, and from *Pinonepalus* by the more strongly convex pronotum in cross-section and the stouter, non-filiform antennae. Additional diagnostic features of *Pinopellis* include the strongly convex pronotum, the dense and conspicuous pubescence near the apex of the abdomen, the presence of anterior impressions on tergites III–VI (the impression on tergite VI is absent only in *P. aspera* Assing, 2022), the derived chaetotaxy of tergite IX, with the long setae arranged in defined, conspicuous tufts, and the pronounced median excision of the anterior margin of the labrum (Assing 2022).

A study of the *Pinopellis* material of China yielded two new species and additional records of *P. biexcisa* Assing, 2022, *P. nigripes* (Cameron, 1914), and *P. yunnanica* Assing, 2022. Additionally, a key of the Chinese species of *Pinopellis* is provided.

## Materials and methods

The genitalia and other dissected parts were mounted on plastic slides and attached to the same pin as the respective specimens. Photographs were taken with a Canon EOS 7D camera with a MP-E 65 mm macro lens or with a Canon G9 camera mounted on an Olympus CX 31 microscope.

Total length (**TL**): length of body from anterior margin of the head to abdominal apex; length of forebody (**FL**): length of forebody from anterior margin of head to posterior margin of elytra; head length (**HL**): length of head from anterior margin of the frons to the posterior margin of the head; head width (**HW**): maximum width of head; antenna length (**AnL**): length of antenna from the base to the apex; pronotum length (**PL**): length of pronotum along midline; pronotum width (**PW**): maximum width of pronotum; elytral length (**EL**): length at suture from apex of scutellum to elytral hind margin; elytral width (**EW**): combined width of elytra; length of aedeagus (**AL**): length of aedeagus from the apex to the base of the aedeagus.

All material treated in this paper is deposited in the Insect Collection of Shanghai Normal University, Shanghai, China (**SNUC**) and Institute of Zoology, Chinese Academy of Sciences, Beijing, China (**IZCAS**). The type labels are cited in the original spelling.

## Results

### Pinopellis biexcisa

Taxon classification

Animalia

ColeopteraStaphylinidae

Assing, 2022

F4A41F13-65DB-5B03-88FA-085CF02F329A

Fig. 1

Pinopellis biexcisa Assing, 2022: 356. Type locality: “BURMA Shan prov., Namhsan 1600 m”.

#### Material examined.

China – **Guangxi** • 1 ♂; Laibin City, Wuxuan County, Shiwushan, 23°29'24"N, 109°52'12"E, alt. 240 m; 17.VIII.2024; Li, Li & Tian leg.; SNUC.

**Figure 1.**
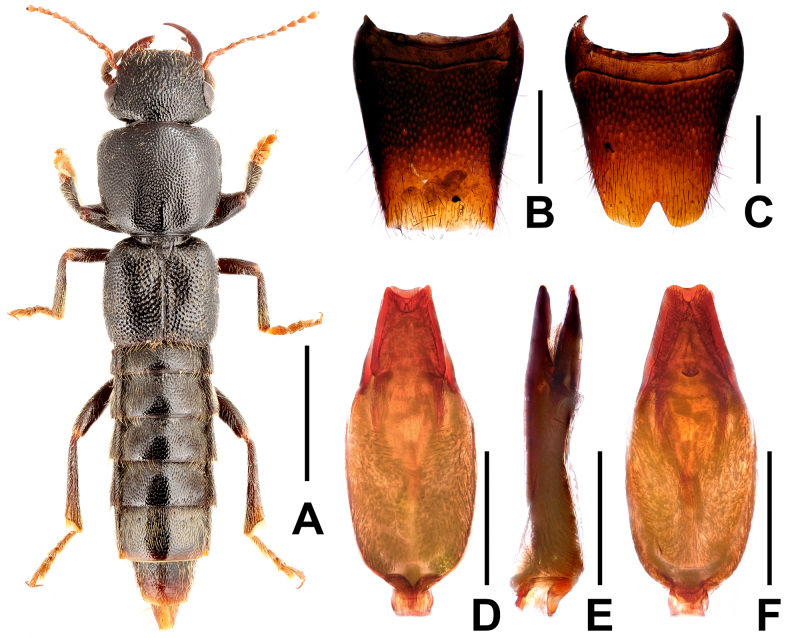
*Pinopellis biexcisa*. **A**. Habitus; **B**. Male tergite VIII; **C**. Male sternite VIII; **D**. Aedeagus in ventral view; **E**. Aedeagus in lateral view; **F**. Aedeagus in dorsal view. Scale bars: 2.0 mm (**A**); 0.5 mm (**B–F**).

#### Comments.

This species was previously known only from Namhsan, Myanmar (Assing 2022). The examined specimen is the first of this species collected from China. It is a male collected in the forest canopy. The identification is confirmed by the morphology of the ventral process and the dorsal plate of the aedeagus, as well as by the measurements of the specimen. For illustrations of *P. biexcisa*, see Fig. 1 and Assing (2022: figs 1100, 1127, 1184, 1185).

### Pinopellis liaoi

Taxon classification

Animalia

ColeopteraStaphylinidae

Huang & Peng sp. nov.

EFDD9FA1-6C87-53C5-A500-5D0AA0D8BA95

https://zoobank.org/2BA079FF-5623-480C-B34D-B69C82A3BA2D

Fig. 2

#### Type material.

***Holotype***. China – **Guangdong Prov**. • ♂; glued on a card with two labels as follows: “China: Guangdong Prov., Shaoguan, Chebaling N. R., 24°43'29"N, 114°15'21"E, 351–460 m; 22.VI.2020; Xia, Zhang, Yin & Lin leg.” “HOLOTYPE: *Pinopellis liaoi* sp. nov., Huang & Peng des. 2026” [red handwritten label]; SNUC. ***Paratype***. China – **Guangdong Prov**. • 1♂; glued on a card with two labels as follows: “China: Guangdong Prov., Nanling, Qinshuigu, leaf litter, 540 m; 21.VII.2008; Gan-yan Yang leg.” “PARATYPE: *Pinopellis liaoi* sp. nov., Huang & Peng des. 2026” [yellow handwritten label]; IZCAS; China – **Guangdong Prov**. • 3♂♂, 2♀♀; glued on a card with two labels as follows: “China: Guangdong Prov., Chebaling N. R., leaf litter, 365 m; 25.VII.2008; Gan-yan Yang leg.” “PARATYPE: *Pinopellis liaoi* sp. nov., Huang & Peng des. 2026” [yellow handwritten label]; IZCAS.

**Figure 2.**
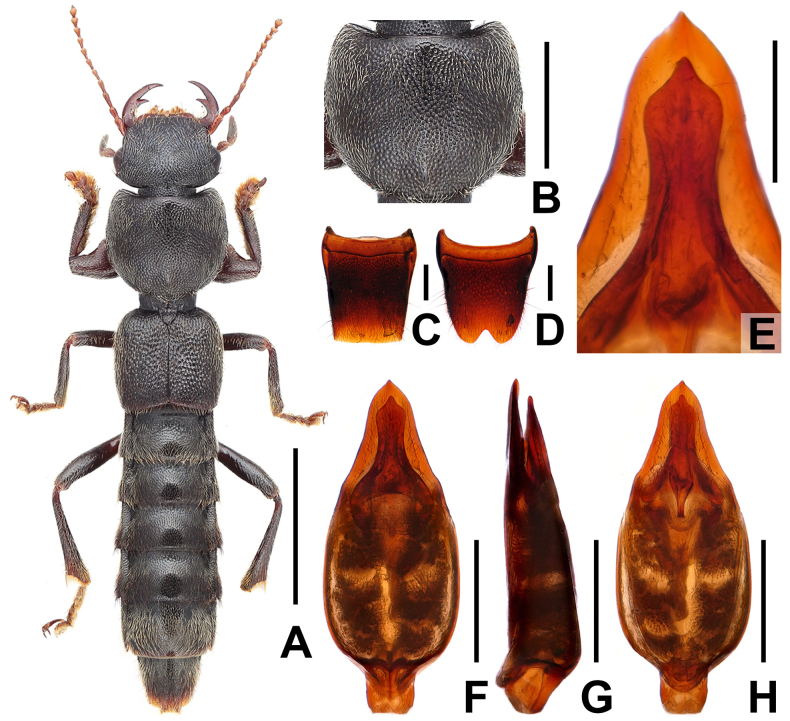
*Pinopellis liaoi*. **A**. Habitus; **B**. Pronotum; **C**. Male tergite VIII; **D**. Male sternite VIII; **E**. Apical portion of aedeagus in ventral view; **F**. Aedeagus in ventral view; **G**. Aedeagus in lateral view; **H**. Aedeagus in dorsal view. Scale bars: 2.0 mm (**A**); 1.0 mm (**B**); 0.5 mm (**C, D, F–H**); 0.2 mm (**E**).

#### Description.

Measurements (in mm) and ratios: TL 9.17, FL 4.89, HL 1.21, HW 1.70, AnL 2.62, PL 1.74, PW 1.97, EL 1.36, AL 1.37, HL/HW 0.71, HW/PW 0.87, HL/PL 0.70, PL/PW 0.88, EL/PL 0.78.

Habitus as in Fig. 2A. Body blackish, abdomen apically paler; legs blackish with dark-brown forelegs; antennae reddish brown.

Head transverse; punctation coarse and very dense; interstices without microsculpture. Eyes strongly convex, 1.45 times as long as postocular region in dorsal view. Antennomeres III–XI slender, of gradually decreasing length and decreasingly oblong; antennomere V 2.44 times as long as broad.

Pronotum (Fig. 2B) moderately transverse, strongly convex in cross-section; posterior angles obsolete; punctation similar to that of head; interstices forming narrow ridges, with shallow microsculpture; midline with rather short, longitudinal, glossy patch posteriorly.

Elytra moderately short; punctation much coarser and sparser than that of pronotum. Hind wings moderately reduced. Protarsomeres 1–4 strongly dilated.

Abdomen parallel, widest at segment VI, with conspicuous dense pale pubescence; tergites III–VI with shallow anterior impressions, these impressions with microsculpture and coarse, dense punctation; remainder of tergites VII–VIII with finer and denser punctation; posterior margin of tergite VII with palisade fringe.

Male. Posterior margin of tergite VIII (Fig. 2C) nearly truncate; sternite VIII (Fig. 2D) transverse, with deeply V-shaped posterior excision; aedeagus stout (Fig. 2E–H); ventral process slender and apically acute, much smaller and narrower than dorsal plate, dilated in apical fourth in ventral view; dorsal plate apically acute.

#### Distribution and natural history.

The type locality is in the Chebaling Natural Reserve northeast of Shaoguan, northern Guangdong. The specimen was sifted from leaf litter in a mixed forest at altitudes of 351–460 m.

#### Etymology.

The species is named after Tao-Kun Liao, who lent extensive support to our study.

#### Comparative notes.

The external and particularly the male sexual characters leave no doubt that this species belongs to the *P. nepalensis* species group (Assing 2022). The aedeagus of *P. liaoi* is most similar to those of *P. yini* sp. nov. and *P. clavulata* Assing, 2022. This species can be distinguished from *P. clavulata* by the smaller body size, the shorter antennae, the larger length-to-width ratio of antennomere V, the more strongly projecting anterior angles of pronotum, the more strongly convex pronotum in cross-section, and the more pronounced lateral projections of the apical portion of the ventral process in ventral view. It is additionally separated from *P. yini* by the smaller body size, the smaller length-to-width ratio of antennomere V, the shorter antennae, the larger ratio of elytral length to pronotal length, the more strongly projecting anterior angles of pronotum, the coarser pronotal punctation, the more strongly convex pronotum in cross-section, the more slender basal portion of the aedeagus, the more acute apex of the ventral process, and the less pronounced lateral projections of the apical portion of the ventral process in ventral view. For illustrations of *P. clavulata*, see Assing (2022: figs 1106, 1133, 1197–1198), and of *P. yini* sp. nov., see Fig. 5.

### Pinopellis nigripes

Taxon classification

Animalia

ColeopteraStaphylinidae

(Cameron, 1914)

0D6B6627-532A-5EA8-8BAD-6851921E701D

Figs 3, 4

Pinophilus nigripes Cameron, 1914: 536. Type locality: “Northern India.”
Pinopellis nigripes Assing, 2022: 337.

#### Material examined.

China – **Xizang** • 1 ♂; Medog County, pass nr. Power Station, 29°16'15"N, 95°12'25"E, alt. 1000 m; 09.VIII.2022; Peng, Song, Yin & Zhang leg.; SNUC • 1 ♀; Medog County, Beibeng Town, 29°15'0"N, 95°10'48"E, alt. 500 m; 10.VIII.2022; Peng, Song, Yin & Zhang leg.; SNUC – **Yunnan** • 1 ♂; Xishuangbanna, Mengla County, Menglun Town, 21°54'21.08"N, 101°16'46.34"E, alt. 575 m; 31.VII.2018; Bai, Chen, Wang & Yu leg. (SNUC); • 1 ♀; Mengla County, Wangtianshu, 21°37'00"N, 101°35'11"E, alt. 655 m; 22.IV.2023; Wang, Lu & Ren leg.; SNUC • 1 ♀; Jinghong, Jinuo Shan, 22°00'N, 100°59'E, alt. 900 m; 31.III.2025; Chen & Peng leg.; SNUC • 1 ♂; Mengla County, Bakaxiaozhai, 21°57'36"N, 101°12'36"E, alt. 700 m; 03.IV.2025; Chen & Peng leg.; SNUC • 1 ♂, 1 ♀; Fugong County, Shiyueliang, 27°08'24"N, 98°30'36"E, 2400–2600 m; 10.XI.2025; Bai-Jun Li leg.; SNUC – **Fujian** • 1 ♂; Sanming County, Chenda Town, Huangcuo Shan, alt. 200 m; 14.IV.2021; Tao-Kun Liao leg.; SNUC • 1 ♂, 1 ♀; Nanjing County, Huboliao, 24°30'58"N, 117°15'45"E, alt. 300 m; 25.VII.2020; Hai-Tian Song leg.; SNUC – **Guangdong** • 1 ♂; Qingyuan County, Qingxin District, Jintan Town, 24°3'20"N, 112°48'29"E, alt. 80 m; 25.V.2025; Xiao Niu leg.; SNUC • 1 ♂, 2 ♀♀; Shaoguan City, Shixing County, Chebaling N. R., 24°43'4.8"N, 114°14'58"E, alt. 395 m; 06.IX.2024; Jiang, Zhang & Liu leg.; SNUC – **Guangxi** • 1 ♂; Yulin City, Rong County, Lantongling, 22°48'50"N, 110°37'44"E, alt. 255 m; 18.VIII.2024; Li, Li & Tian leg.; SNUC.

**Figure 3.**
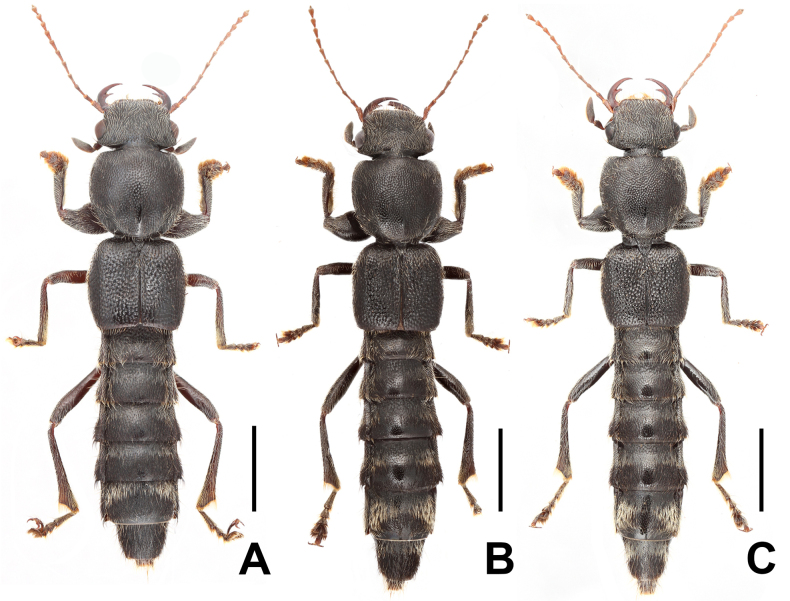
Habitus of *Pinopellis nigripes*. **A**. From Fujian; **B**. From Yunnan; **C**. From Xizang. Scale bars: 2.0 mm.

**Figure 4.**
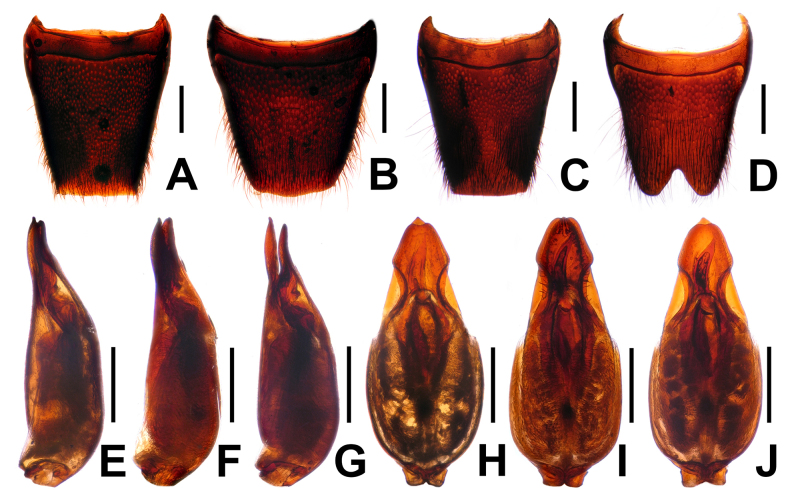
*Pinopellis nigripes*. **A**. Female tergite VIII; **B**. Female sternite VIII; **C**. Male tergite VIII; **D**. Male sternite VIII. Aedeagus of *P. nigripes* from different provinces; **E–G**. In lateral view and **H–J**. In ventral view. **E, H**. From Fujian; **F, I**. From Yunnan; **G, J**. From Xizang. Scale bars: 0.5 mm.

#### Comments.

*Pinopellis nigripes* is widespread from eastern Nepal and northern India across southern China, Thailand, Laos, Vietnam, and Peninsular Malaysia to some of the Indonesian Sunda Islands (Assing 2022). This species is subject to considerable intraspecific variation, such as punctation of the head and pronotum (Fig. 3) and the shape of the apical portion of the dorsal plate of the aedeagus (Figs 4E–J). However, the body proportions, especially the HW/PW, PL/PW, and EL/PL ratios, as well as the broader ventral process relative to the dorsal plate, appear to be stable within the species. The material from Fujian, Guangdong, Guangxi, and Xizang represents new provincial records. For illustrations of *P. nigripes*, see Figs 3, 4, as well as Assing (2022: figs 1085–1086, 1110–1111, 1146–1151).

### Pinopellis yini

Taxon classification

Animalia

ColeopteraStaphylinidae

Huang & Peng sp. nov.

5644BA77-F698-50B4-8044-E2AEC9E2CD57

https://zoobank.org/1539D327-4E3C-4A40-96B9-E6326403E28B

Fig. 5

#### Type material.

***Holotype***. China – **Xizang Prov**. • ♂; glued on a card with two labels as follows: “China: Xizang Prov., Medog County, Beibeng, road to Gelin Vil., 29°14'49"N, 95°11'03"E, 1060 m; 23.VII.2019; Zi-Wei Yin leg.” “HOLOTYPE: *Pinopellis yini* sp. nov., Huang & Peng des. 2026” [red handwritten label]; (SNUC).

**Figure 5.**
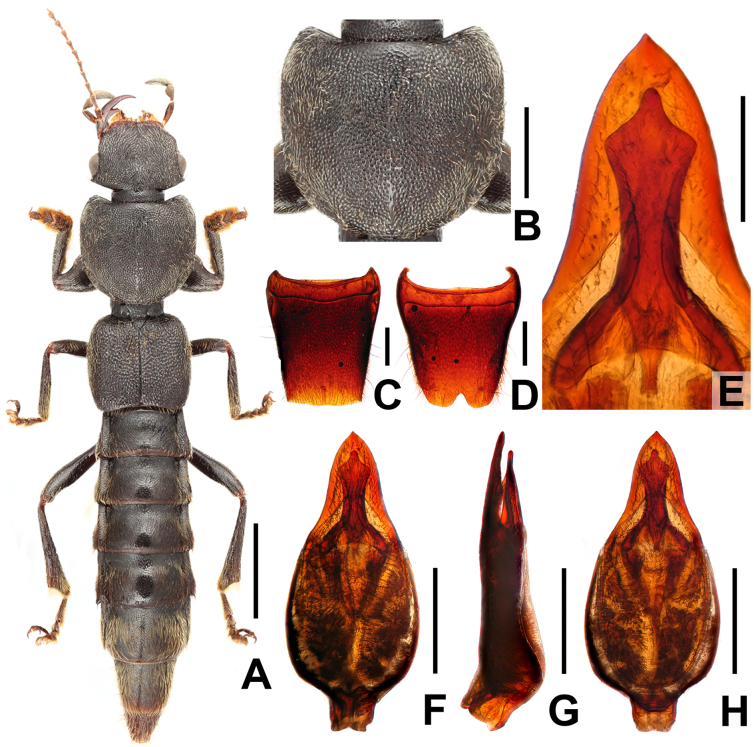
*Pinopellis yini*. **A**. Habitus; **B**. Pronotum; **C**. Male tergite VIII; **D**. Male sternite VIII; **E**. Apical portion of aedeagus in ventral view; **F**. Aedeagus in ventral view; **G**. Aedeagus in lateral view; **H**. Aedeagus in dorsal view. Scale bars: 2.0 mm (**A**); 1.0 mm (**B**); 0.5 mm (**C**, **D**, **F**–**H**); 0.2 mm (**E**).

#### Description.

Measurements (in mm) and ratios: TL 13.01, FL 6.12, HL 1.44, HW 2.08, AnL 3.41, PL 2.19, PW 2.46, EL 1.51, AL 1.41, HL/HW 0.69, HW/PW 0.85, HL/PL 0.66, PL/PW 0.89, EL/PL 0.69.

Habitus as in Fig. 5A. Body blackish; abdomen apically paler; legs blackish; antennae and maxillary palpi reddish brown.

Head transverse; punctation moderately coarse and very dense; interstices without microsculpture. Eyes strongly convex, 1.76 times as long as postocular region in dorsal view. Antennomeres III–XI slender, of gradually decreasing length and decreasingly oblong; antennomere V 2.73 times as long as broad.

Pronotum (Fig. 5B) moderately transverse, strongly convex in cross-section; posterior margins forming smoothly convex outline, posterior angles obsolete; punctation similar to that of head; interstices forming narrow ridges, with microsculpture; midline with moderately short, longitudinal, glossy patch posteriorly.

Elytra moderately short; punctation much coarser and sparser than that of pronotum; interstices without microsculpture. Hind wings moderately reduced. Protarsomeres 1–4 strongly dilated.

Abdomen parallel, widest at segment VI; tergites III–VI with shallow anterior impressions, these impressions with microsculpture and moderately coarse, sparse punctation; remainder of tergites III–VIII with dense and fine punctation; tergites VII–VIII with conspicuous dense pale pubescence; posterior margin of tergite VII with palisade fringe.

Male. Posterior margin of tergite VIII (Fig. 5C) weakly convex; sternite VIII (Fig. 5D) weakly transverse, with small V-shaped posterior excision; aedeagus stout (Figs 5E–H), ventral process slender, subapically moderately dilated and rounded apically, much smaller and narrower than dorsal plate; dorsal plate apically acute.

#### Distribution and natural history.

The type locality is situated in Gelin to the southwest of Medog, southeastern Xizang. The specimen was sifted from leaf litter in shrub habitats at an altitude of 1060 m.

#### Etymology.

The species is named after Zi-Wei Yin, who is the collector of the type specimen.

#### Comparative notes.

As can be inferred from the male sexual characters (shape of the ventral process of the aedeagus in ventral view; shape and chaetotaxy of the male sternite VIII), *P. yini* belongs to the *P. nepalensis* species group (Assing 2022). This species is closely allied to *P. liaoi* sp. nov. and *P. clavulata* Assing, 2022. It differs from *P. clavulata* by the larger length-to-width ratio of antennomere V, the longer antenna, the more strongly projecting anterior angles of the pronotum, the finer pronotal punctation, the stouter basal portion of the aedeagus, the more rounded apex of the ventral process, and the more pronounced lateral projections of the apical portion of the ventral process in ventral view. It is additionally separated from *P. liaoi* by the larger body size, the larger length-to-width ratio of antennomere V, the longer antennae, the smaller ratio of elytral length to pronotal length, the less strongly convex pronotum in cross-section, the finer pronotal punctation, the stouter basal portion of the aedeagus, the more rounded apex of the ventral process, and the more pronounced lateral projections of the apical portion of the ventral process in ventral view. The aedeagus of *P. yini* is also similar to those of *P. coarcticollis* (Cameron, 1941) and *P. spiculata* Assing, 2022; however, these two species are geographically remote and differ in the punctation of the head and, therefore, belong to a different species group. For illustrations of *P. clavulata*, see Assing (2022: figs 1106, 1133, 1197–1198), and of *P. liaoi* sp. nov., see Fig. 2.

### Pinopellis yunnanica

Taxon classification

Animalia

ColeopteraStaphylinidae

Assing, 2022

B320B493-FD85-54BE-AB18-57FA87DDB881

Fig. 6

Pinopellis yunnanica Assing, 2022: 358. Type locality: “S. CHINA, Yunnan prov., Xiquanbanna, Lancang riv. Vall., 15 km N. Jinghong, 800 m.”

#### Material examined.

China – **Guangdong** • 3 ♂♂, 3 ♀♀; Qingyuan County, Qingxin District, Jintan Town, Huangqitang Village, 24°3'20"N, 112°48'29"E, alt. 80 m; 25.V.2025; Xiao Niu leg.; SNUC – **Yunnan** • 1 ♂; Fugong County, Shiyueliang, 27°8'24"N, 98°30'36"E, alt. 2500 m; Gu Gu leg.; SNUC • 1 ♂; Yuxi, Eshan, Yubaiding, Green Peafowl Reserve; 10.VI.2025; Zhen-Bang Xu leg.; SNUC.

**Figure 6.**
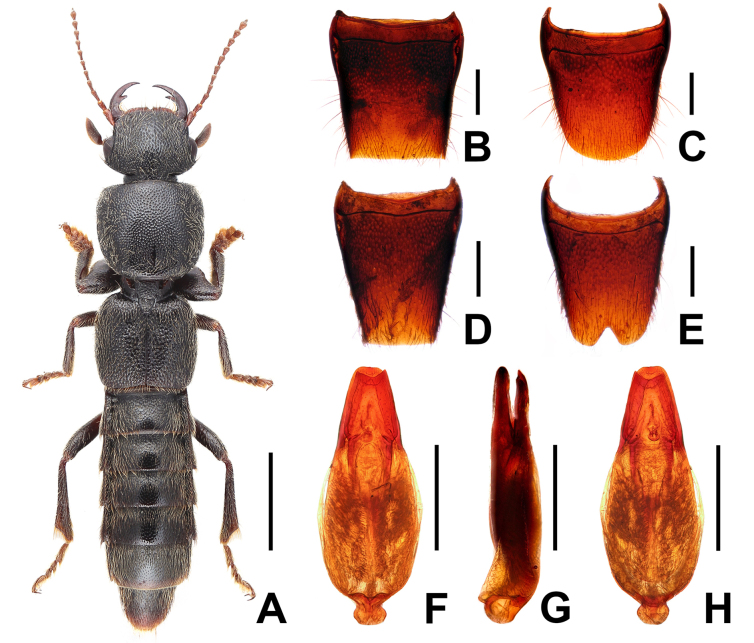
*Pinopellis yunnanica*. **A**. Habitus; **B**. Female tergite VIII; **C**. Female sternite VIII; **D**. Male tergite VIII; **E**. Male sternite VIII; **F**. Aedeagus in ventral view; **G**. Aedeagus in lateral view; **H**. Aedeagus in dorsal view. Scale bars: 2.0 mm (**A**); 0.5 mm (**B–H**).

#### Comments.

In both external and the male sexual characters, this species is similar to *P. biexcisa* Assing, 2022 and *P. truncata* Assing, 2022. It can be distinguished from *P. biexcisa* by the more truncate apex of the dorsal plate of the aedeagus. It is additionally separated from *P. truncata* by the longer ventral process of the aedeagus. This species was only recently described from Yunnan Province (Assing 2022). The above material from Guangdong represents new provincial records. For illustrations of *P. yunnanica*, see Fig. 6 and Assing (2022: figs 1103, 1130, 1191–1193).

### Pinopellis

Taxon classification

Animalia

ColeopteraStaphylinidae

sp. indet. 1

992A5501-0FF9-51D9-A048-7994BF6EAE13

Fig. 7A, C–F, I–K

#### Material examined.

China – **Hainan** • 1 ♂; Jianfengling N. R., Mingfenggu Valley, 18°44'N, 108°50'E, 950 m; 29.IV.2012; Peng & Dai leg.; SNUC • 1 ♂, 1 ♀; Lingshui County, Diaoluo Shan, Winding Road, 18°42'N, 109°52'E, 600–1000 m; 26.IV.2012; Peng & Dai leg.; SNUC.

**Figure 7.**
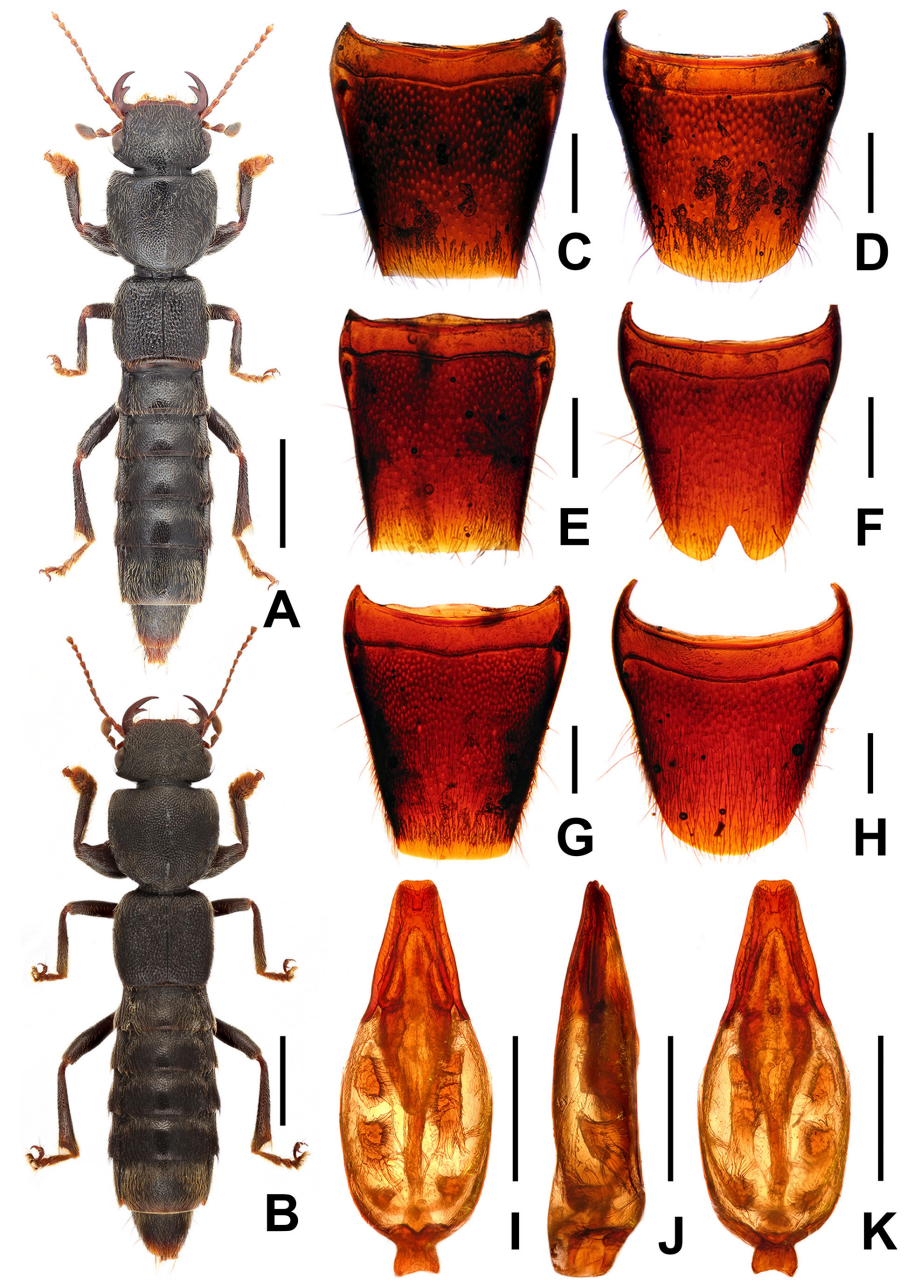
**A, C–F, I–K**. *Pinopellis* sp. indet. 1; **A**. Habitus; **C**. Female tergite VIII; **D**. Female sternite VIII; **E**. Male tergite VIII; **F**. Male sternite VIII; **I**. Aedeagus in ventral view; **J**. Aedeagus in lateral view; **K**. Aedeagus in dorsal view; **B, G, H**. *P.* sp. indet. 2; **B**. Habitus; **G**. Female tergite VIII; **H**. Female sternite VIII. Scale bars: 2.0 mm (**A, B**), 0.5 mm (**C–K**).

#### Comments.

The Hainan specimens were provisionally assigned to *Pinopellis linmeiyingae* Li et al., 2026. The holotype of *P. linmeiyingae* was collected from Beibeng, Medog, Xizang, and the paratype from Mingfenggu Valley, Jianfengling, Ledong County, Hainan. The specimens examined in this study were obtained from Diaoluoshan and Jianfengling, Hainan, at altitudes of 600–1000 m. The Jianfengling specimen originates from the paratype locality, and the Diaoluoshan locality is approximately 100 km from the latter. However, the measurements of the Hainan specimens differ from those reported for *P. linmeiyingae*. The body size and length of aedeagus of the Hainan specimens (TL = 9.29 mm, FL = 4.78 mm, AL = 1.31–1.37 mm) are markedly smaller than those reported by Li et al. (2026) for *P. linmeiyingae* (TL = 10.7–13.2 mm, FL = 6.33–6.36 mm, AL = 1.48 mm). In addition, they differ in the shape of the apex of the aedeagus. Moreover, the holotype illustrated in the original publication is from Xizang, whereas no material from Xizang was available for direct comparison in the present study; only specimens from Hainan were examined. Since the species is brachypterous, it cannot be excluded that the populations from Xizang and Hainan represent different (cryptic) species or local races. In addition, these specimens are similar to *P. yunnanica*, but this unidentified species can be distinguished from it by the longer and paler antennae, the smaller ratio of elytral length to pronotal length, and the more slender and symmetric ventral process of the aedeagus.

### Pinopellis

Taxon classification

Animalia

ColeopteraStaphylinidae

sp. indet. 2

1B11C2ED-DCF2-5463-976E-41686EEBC333

Fig. 7B, G, H

#### Material examined.

China – **Guizhou** • 1 ♀; Rongjiang County, Xiaodanjiang, 26°20'16.09"N, 108°20'23.34"E, alt. 700 m; 05.V.2021; Tang, Peng, Cai & Song leg.; SNUC.

#### Comments.

The female specimen from Guizhou is possibly referable to *P. clavulata* Assing, 2022. According to Assing (2022), the holotype male of *P. clavulata* was collected in Jinggangshan, Jiangxi, whereas the Guizhou material consists only of female specimens. The specimen examined in the present study is regarded as conspecific with the Guizhou specimen reported by Assing. The two localities are only about 100 km apart and the measurements of this female specimen (TL = 12.12 mm, FL = 6.06 mm, HW/PW = 0.85, PL/PW = 0.91, EL/PL = 0.74) are similar to those reported by Assing (TL = 12–13 mm, FL = 5.7–6.3 mm, HW/PW = 0.88–0.89, PL/PW = 0.88–0.89, EL/PL = 0.71–0.75). However, in Assing (2022), the two localities in Jiangxi and Guizhou are approximately 650 km apart. Moreover, the hind wings of this species were described as probably of reduced length. In view of the considerable geographic distance between the Jiangxi and Guizhou localities and the presumably limited dispersal ability implied by the reduced hind wings, it cannot be excluded that the Jiangxi and Guizhou specimens may represent different species. In the absence of the male specimen for confirmation, this specimen cannot be assigned to *P. clavulata* with certainty at present.

##### Key to *Pinopellis* species of China

**Table:** 

1	Hind wings well developed; tergites III–VI with broad, distinct anterior impression; aedeagus length more than 1.75 mm	***P. nigripes* (Cameron, 1914)**
–	Hind wings more or less reduced; tergites III–VI with shallow, inconspicuous anterior impression; aedeagus length no more than 1.50 mm	**2**
2	Ventral process of aedeagus apically acute or rounded, and distinctly shorter than dorsal plate	**3**
–	Ventral process of aedeagus apically concave, and nearly as long as dorsal plate	**5**
3	Antenna longer, length more than 3.4 mm, antennomere V more than 2.70 times as long as broad; ventral process of aedeagus apically rounded	***P. yini* Huang & Peng, sp. nov**.
–	Antenna shorter, length no more than 2.8 mm, antennomere V no more than 2.45 times as long as broad; ventral process of aedeagus apically acute	**4**
4	Smaller species, length of body no more than 9.2 mm, length of forebody no more than 5.0 mm; antennomere V more than 2.4 times as long as broad; aedeagus length no more than 1.38 mm	***P. liaoi* Huang & Peng, sp. nov**.
–	Larger species, length of body more than 12.0 mm, length of forebody more than 6.0 mm; antennomere V no more than 2.2 times as long as broad; aedeagus length more than 1.48 mm	***P. clavulata* Assing, 2022**
5	Length of forebody more than 6.0 mm; pronotum with an almost complete impunctate middle line; EL/PL more than 0.9; aedeagus length more than 1.45 mm	***P. linmeiyingae* Li et al., 2026**
–	Length of forebody no more than 5.3 mm; pronotum with a reduced impunctate middle line only in posterior portion; EL/PL no more than 0.8; aedeagus length no more than 1.32 mm	**6**
6	Antennae brown to dark brown; maxillary palpi black; aedeagus longer (1.30 mm), dorsal plate more strongly concave apically	***P. yunnanica* Assing, 2022**
–	Antennae reddish; maxillary palpi blackish brown; aedeagus shorter (1.25 mm), with dorsal plate weakly concave apically	***P. biexcisa* Assing, 2022**

## Supplementary Material

XML Treatment for Pinopellis biexcisa

XML Treatment for Pinopellis liaoi

XML Treatment for Pinopellis nigripes

XML Treatment for Pinopellis yini

XML Treatment for Pinopellis yunnanica

XML Treatment for Pinopellis

XML Treatment for Pinopellis

## Additional information

### Artificial Intelligence (AI) use

The authors accept full responsibility for the content of the manuscript, including the disclosure of any use of AI.

No AI tools were used in the preparation of this manuscript.

### Author ORCIDs

L. Huang https://orcid.org/0009-0007-3265-0832

Z. Peng https://orcid.org/0000-0001-5959-1536

### Data availability

All of the data that support the findings of this study are available in the main text.
